# Identification of prognostic alternative splicing events in sarcoma

**DOI:** 10.1038/s41598-021-94485-x

**Published:** 2021-07-22

**Authors:** Hongshuai Li, Jie Yang, Guohui Yang, Jia Ren, Yu Meng, Peiyi Qi, Nan Wang

**Affiliations:** 1 Department of Emergency Surgery, The First Affiliated Hospital of Zhengzhou University, Zhengzhou, 450052 China; 2 Department of Operation, The First Affiliated Hospital of Zhengzhou University, Zhengzhou, 450052 China

**Keywords:** Sarcoma, Tumour biomarkers

## Abstract

Sarcoma is a rare malignancy with unfavorable prognoses. Accumulating evidence indicates that aberrant alternative splicing (AS) events are generally involved in cancer pathogenesis. The aim of this study was to identify the prognostic value of AS-related survival genes as potential biomarkers, and highlight the functional roles of AS events in sarcoma. RNA-sequencing and AS-event datasets were downloaded from The Cancer Genome Atlas (TCGA) sarcoma cohort and TCGA SpliceSeq, respectively. Survival-related AS events were further assessed using a univariate analysis. A multivariate Cox regression analysis was also performed to establish a survival-gene signature to predict patient survival, and the area-under-the-curve method was used to evaluate prognostic reliability. KOBAS 3.0 and Cytoscape were used to functionally annotate AS-related genes and to assess their network interactions. We detected 9674 AS events in 40,184 genes from 236 sarcoma samples, and the 15 most significant genes were then used to construct a survival regression model. We further validated the involvement of ten potential survival-related genes (*TUBB3*, *TRIM69*, *ZNFX1*, *VAV1*, *KCNN2*, *VGLL3*, *AK7*, *ARMC4*, *LRRC1*, and *CRIP1*) in the occurrence and development of sarcoma. Multivariate survival model analyses were also performed, and validated that a model using these ten genes provided good classifications for predicting patient outcomes. The present study has increased our understanding of AS events in sarcoma, and the gene-based model using AS-related events may serve as a potential predictor to determine the survival of sarcoma patients.

## Introduction

Sarcomas are rare and recalcitrant malignant mesenchymal tumors represented by more than 100 subtypes with different prognoses^1^. Sarcomas account for 1–3% of all malignancies and estimates suggest that approximately 15,610 new cases, and 6480 deaths, occur every year in the United States^2^. Sarcoma is often characterized by being occult, being difficult to diagnose early, having early and distant metastases, and having an overall 5-year survival rate of less than 15% for advanced-stage disease^3^. Despite the therapeutic advances being made, the heterogeneity of sarcomas makes them difficult to diagnose and their treatments complex and multidisciplinary. Therefore, a more in-depth understanding of sarcoma oncogenesis is essential to elucidate the underlying oncogenic mechanisms and to identify novel diagnostic and therapeutic biomarkers for sarcoma patients.

Accumulating evidence has demonstrated that aberrant regulation of gene expression is involved in sarcoma initiation and tumor progression^4^. Alternative splicing (AS) is a ubiquitous and important regulatory step in gene expression, enabling a fixed number of genes to generate a large variety of mature mRNAs. This increased transcript variety is also responsible for expanding the diversity of the transcriptome and the proteome. AS also has the potential to regulate gene expression, and therefore impact important molecular events for cellular differentiation and cell-type specific functions^5^. In the human genome, AS has been shown to play a vital role in the regulation of multiple molecular mechanisms and biological functions^6^. Aberrant AS events results in the inactivation of tumor-suppressing genes, promotion of angiogenesis, acceleration of proliferation, and inhibition of apoptosis^7^. Prominent in cancer, AS has now become a potential target for cancer therapeutics^8^. AS events are post-transcriptional processes that modulate gene expression, and are responsible for the increased proteomic diversity seen in a variety of cancers activities^4,9^. Aberrant AS events may also be relevant for explaining functional transformations in cancers^7,10^, often referred to as the "hallmarks of cancer" for prostate, ovarian, breast, colon, bladder, and lung cancer, among others^11–17^. AS events have also been reported to be involved in the regulation of apoptosis and autophagy genes^18,19^, tumor responses to chemotherapy, the orchestration of cancer stem-cell biology, and in epithelial-to-mesenchymal tumor transitions^7,20,21^. The regulators of RNA AS have the potential to become novel oncoproteins, contributing to dysregulation by modulating RNA isoforms in cancer-related pathways, and may be both biomarkers and targets for sarcoma diagnostics, prognostics, and treatments.

The Cancer Genome Atlas (TCGA) database contains a large number of gene profiles that can be used to investigate novel AS-related gene expression and prognosis data for cancer. In this study, we systematically analyzed AS events and constructed an AS-based gene model based on the sarcoma database in TCGA.

## Results

### The sarcoma landscape of AS events

**Figure 1:**
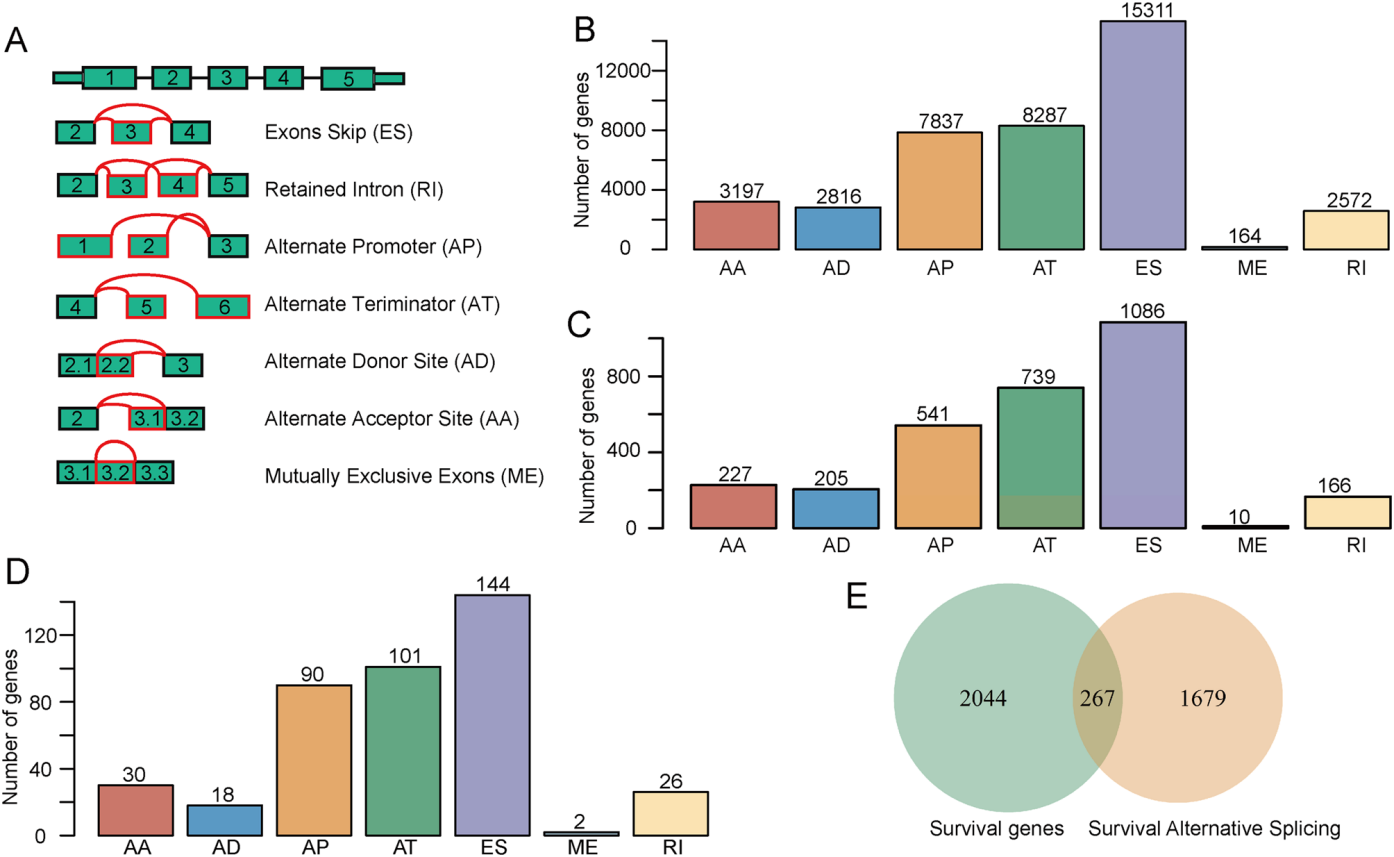
An overview of AS events in sarcoma. (**A**) The seven different subtypes of AS classification include ES, RI, AP, AT, AD, AA, and ME. (**B**) The relationship between AS events and their distribution among the seven subtypes. The ES events occurred in 15,311 genes, whereas the AT and AP events occurred in 8287 and 7837 genes, respectively. (**C**) Histogram showing the distribution among the seven types of splicing events that were significantly associated with overall survival prognoses. The ES, AT, and AP events accounted for the majority of the splicing events associated with overall survival prognoses. (**D**) Histogram showing the distribution among the seven types of splicing events that were associated with survival-related genes. The ES, AT and AP events presented a large amount of survival related genes. (**E**) Venn diagram showing the intersection between survival-related AS and survival-related genes. In total, we identified 267 genes related to survival.

AS-event profiles were identified and analyzed in-depth from 236 sarcoma samples from the TCGA sarcoma cohort. We identified the seven AS patterns (ES, ME, RI, AP, AT, AD, and AA) listed in Fig. 1A. In total, we detected 9674 AS events in 40,184 genes, as illustrated in Fig. 1B. The ES pattern (15,311) was the most frequent AS-subtype, accounting for almost one-third of all events, followed by AT (8287), AP (7837), AA (3197), AD (2816), RI (2572), and ME events (164). Collectively, our findings demonstrate that these seven AS subtypes are frequently involved in sarcoma.

### Prognosis-associated AS events

To detect AS-event gene frequencies in sarcoma and survival, we integrated the clinical survival data from sarcoma patients. Using a univariate survival analysis, we obtained 2974 significant associations between prognoses and AS events (Fig. 1C), and 2311 significant associations between AS events and survival-related genes (Fig. 1D). We determined that 267 of these genes intersected between the AS-event cohort and the survival cohort (Fig. 1E). These results indicate that the majority of ES, AT, and AP events are significantly associated not only with overall prognoses for survival but also with survival genes.

### AS-event subtypes indicate different prognoses

**Figure 2:**
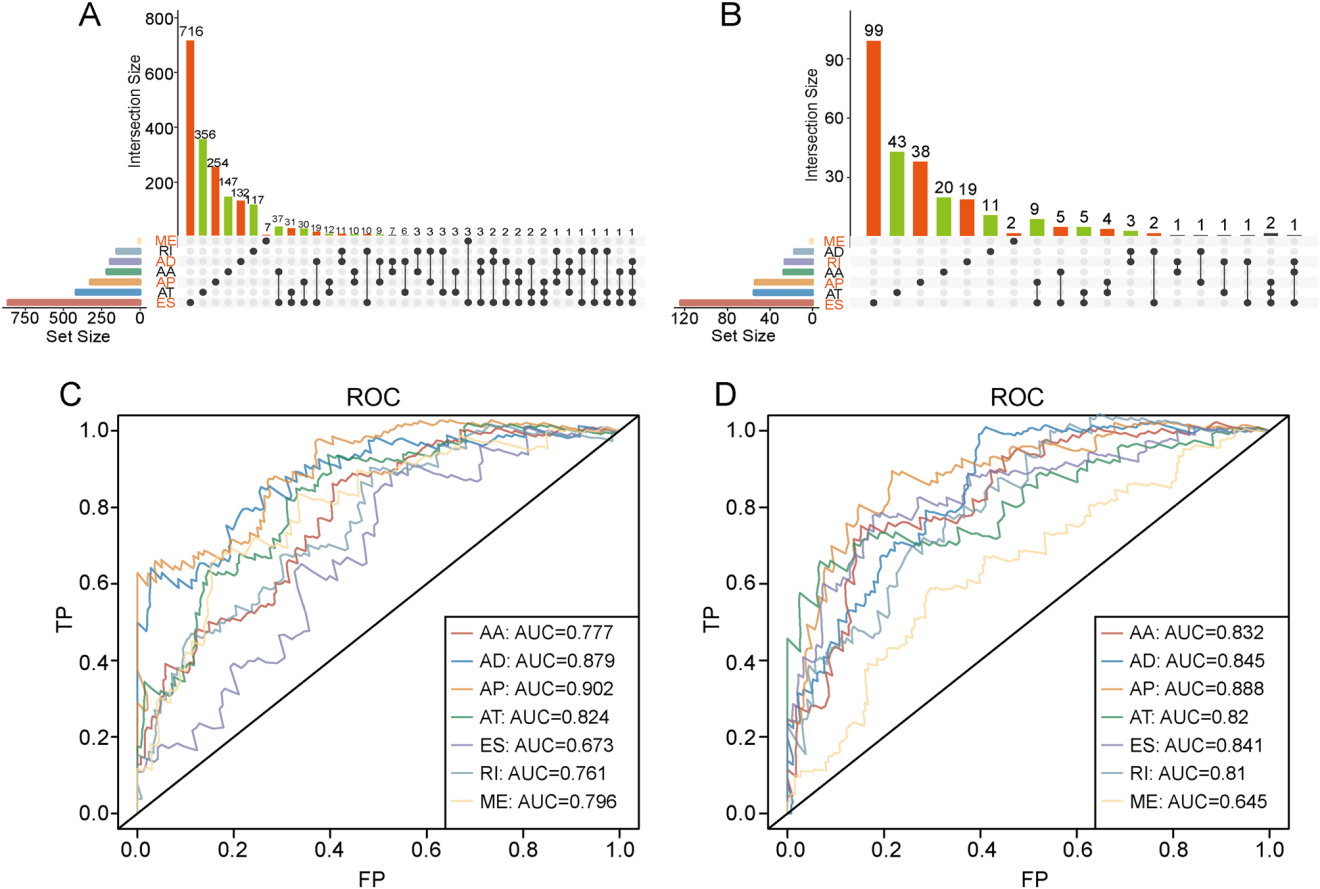
AS events are closely related to sarcoma prognoses. (**A**) The landscape of the seven subtypes of AS-associated genes that were significantly related to overall survival. (**B**) Distribution among the seven subtypes of AS-associated genes that were significantly related to survival genes. (**C**) AS-subtype area-under-the-curve (AUC) analyses for the classification of the top 15 AS-associated genes based on prognoses. (**D**) AS-subtype AUC analyses for prognosis classifications of the top 15 AS-associated genes based on multivariate modeling.

To determine the prognoses associated with the seven different AS-event subtypes, we calculated the relationships between AS subtypes and the landscape for overall survival genes. The seven AS-event subtypes were closely associated with overall survival prognoses (Fig. 2A), and an analysis of genes significantly related to AS events indicated that single genes could have a variety of AS events that are significantly associated with patient survival (Fig. 2B).

### AS-related factors for prognosis predictions

To determine possible prognostic factors based on sarcoma AS events, we selected the 15 most significant genes to construct a survival regression model using area-under-the-curve (AUC) values. The results demonstrated that the seven AS-event subtypes all had high AUC values. For overall survival, the AUC values showed that AP (AUC = 0.902) was highly accurate and that AD (AUC = 0.879) and AT (AUC = 0.824) were well-suited for prognostic predictions. The other subtypes (AA: AUC = 0.777; RI: AUC = 0.761; ME: AUC = 0.796; and ES: AUC = 0.673) also had predictive AUC values (Fig. 2C), suggesting that AS-event subtypes associated with overall survival could serve as novel markers for prognostic predictions. The AP subtype also performed best in the variable-splicing associated with these 15 survival genes (Fig. 2D), indicating that analyses of AS events could be used as a new prognostic classification method for sarcoma.

### AS event related gene interaction network construction

**Figure 3:**
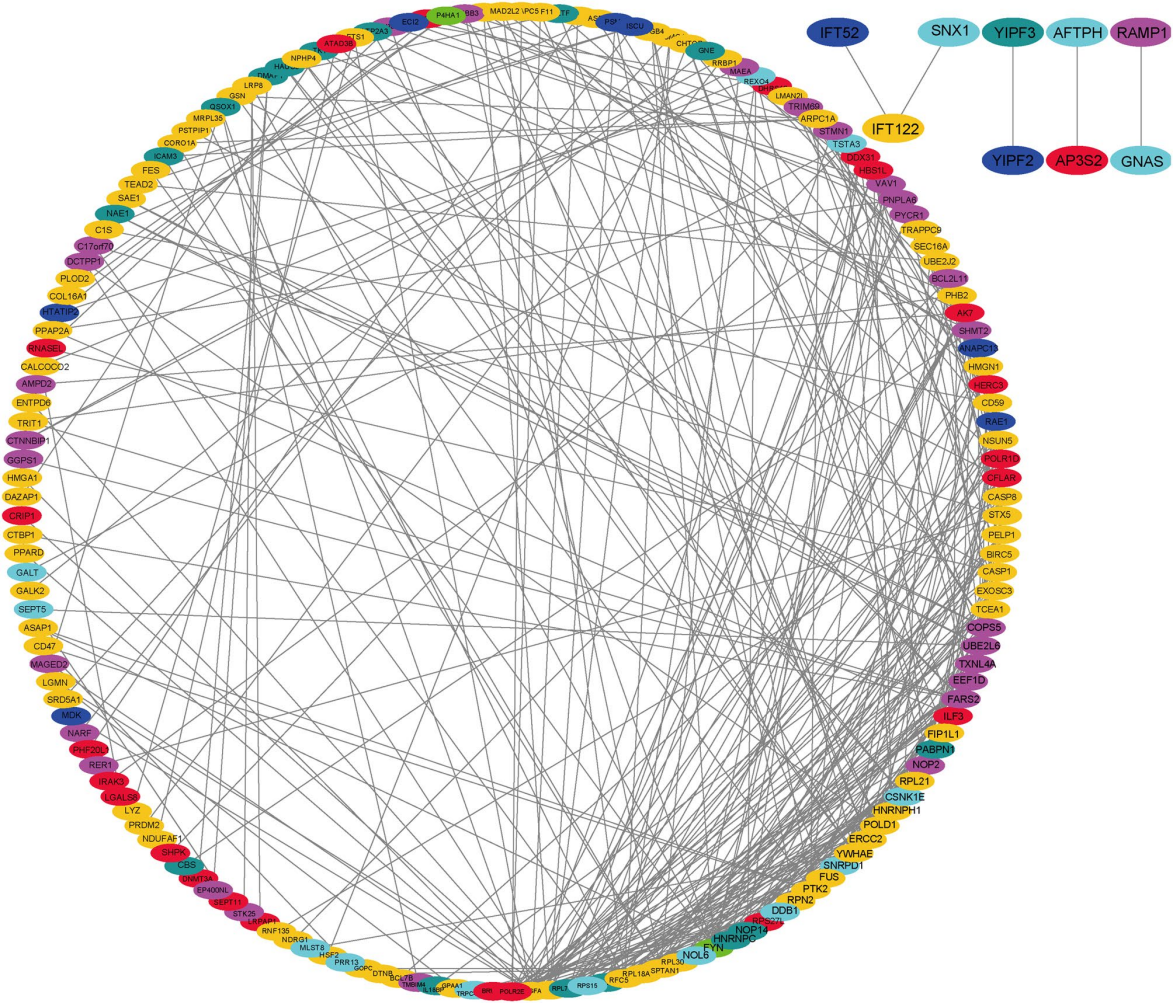
The gene interaction network significantly related to overall survival. The different colors correspond to different AS-event subtypes, where yellow represents ES events and red represents ES events, according to the annotation on diagram. The ES event related genes, such as VEGFA, NME1, PTK2, and RFC5, showed significant interactions with the 267 genes associated with overall survival prognoses.

To further explore the interactions between survival-related AS events in sarcoma, we constructed a STRING database network using score values > 0.4. The 267 significantly associated overall survival genes, for the seven AS-event subtypes, were illustrated in Fig. 3. Using the protein–protein network construction for the corresponding genes involved in the regulatory network for each AS-event, the analysis showed that the AD and RI subtypes had the majority of interactions. The results also demonstrated that the prognosis-related genes associated with variable-splicing events had protein–protein interactions, with most of these genes involved in different biological functions.

### Gene functional analysis

**Figure 4:**
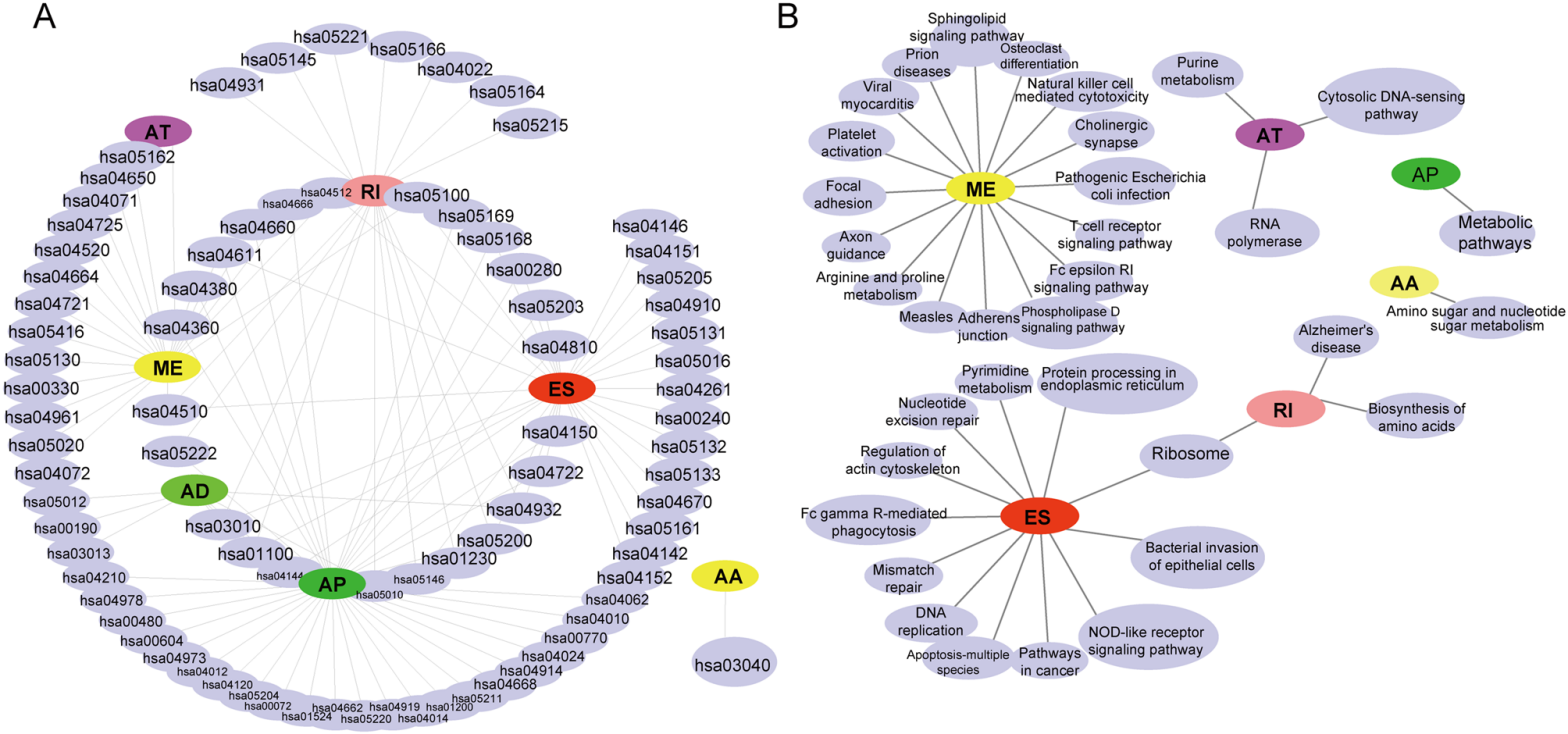
Functional annotations of AS events. (**A**) A KEGG enrichment analysis of the genes that were significantly related to overall survival. The AS events, such as ES, AP, RI, and ME, were the top 4 AS events that showed significant correlation with genes related to overall survival prognoses. (**B**) The KEGG enrichment results for the AS genes with the highest values for gene significance. AS events are closed linked to various biological functions. The results indicated that the ES and ME events are involved in complex pathway interactions, such as those related to nuclear, cancer, and immune cell pathways, among others.

To investigate the gene functions involved in the different types of variable-splicing events that were significantly related to patient prognoses, we performed a KEGG pathway-enrichment analysis^22^ (Fig. 4A). The ES and ME subtypes were involved in a variety of pathways. The ME subtype was correlated with platelet activation, focal adhesion, arginine and proline metabolism, and axon guidance, among others. The ES subtype was correlated with the regulation of the actin cytoskeleton, nucleotide excision repair, mismatch repair, and DNA replication, among others. The RI subtype was involved with ribosomes, the biosynthesis of amino acids, and Alzheimer’s disease, and the AP subtype was involved in metabolic pathways. The AA subtype was involved in amino-sugar and nucleotide-sugar metabolisms, and the AT subtype was involved in purine metabolism, cytosolic DNA-sensing pathways, and with RNA polymerase (Fig. 4B). These genes were also enriched in multiple disease-related pathways, suggesting their involvement in a multitude of biological functions through these seven subtypes of AS events that are significantly related to patient survival.

### Gene expression profiling and prognosis

To determine any correlations between gene expression and the prognoses for these variable-splicing events, that were significantly related to patient outcomes, we utilized a univariate survival analysis based on TCGA RNA-seq gene expression data. From this data, we used the 267 survival-related AS genes that were correlated with patient prognoses, and then Pearson correlations were performed to assess significance. We found that 149 (55.81%) of these genes were significantly related to variable-splicing (*P* < 0.05), indicating that the variable-splicing events of most genes were significantly related to their expression levels.

### Hub gene selection

**Figure 5:**
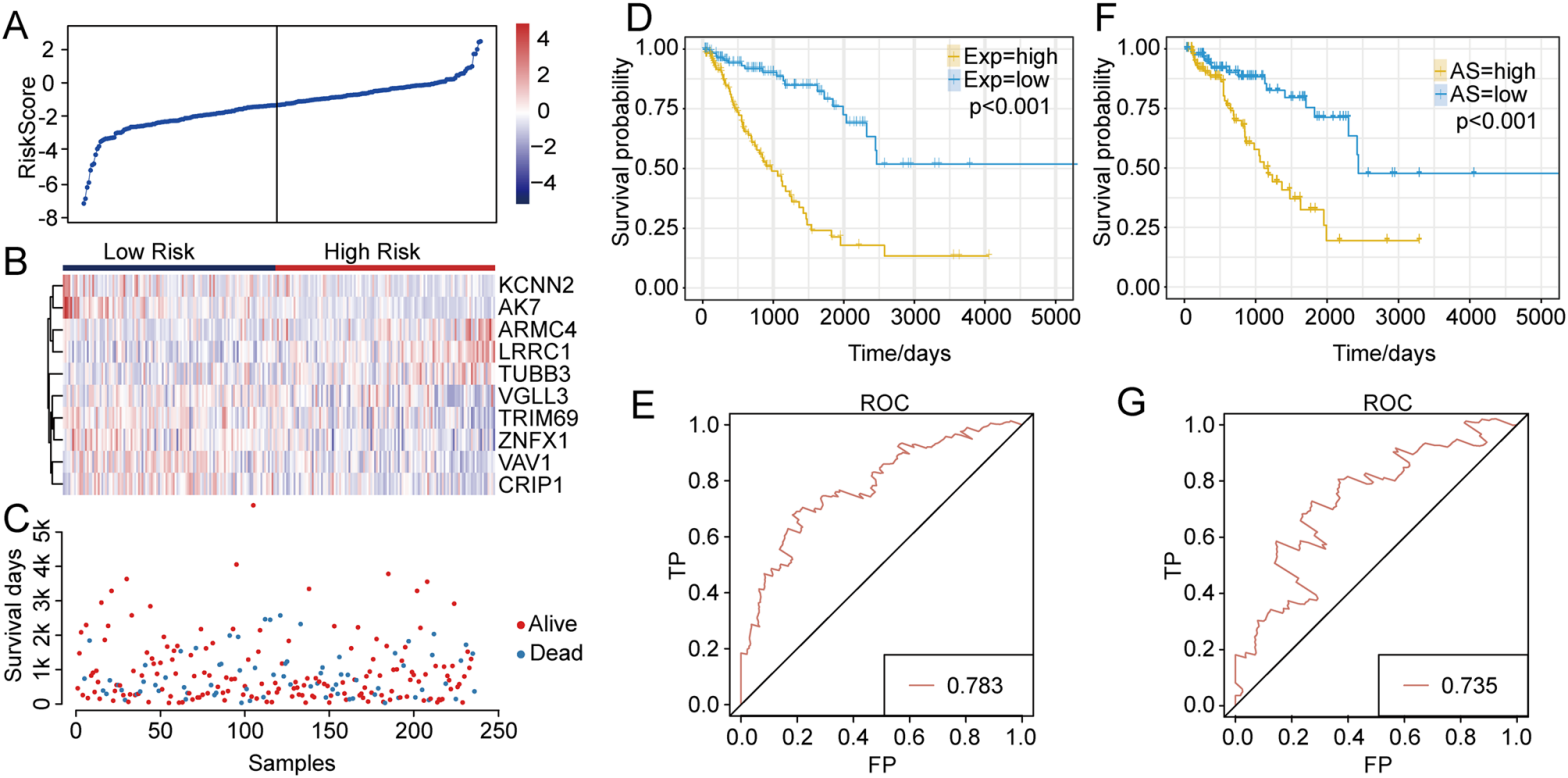
AS gene-related risk scores and survival analyses. (**A**) Risk-score analysis of the sarcoma samples. (**B**) The gene expression heat map for the ten hub genes. Gene expression differences were significant between the high- and low-risk groups. (**C**) Relationship between survival time and survival status for each sarcoma sample. (**D**) Kaplan–Meier curves for overall survival and transcriptome gene expression levels. (**E**) Area-under-the-curve (AUC) multifactorial survival analysis related to overall patient survival based on gene expression at the transcriptome level. (**F**) Kaplan–Meier curves for overall survival and AS genes. (**G**) AUC multifactorial analysis of AS gene overall survival related to patient survival.

To determine the hub genes associated with AS events, we identified 21 genes with expression levels and AS-related Pearson correlation coefficients with absolute values > 0.5. A Cox multivariate analysis was then performed to identify independent prognostic factors. Using these methods, we identified ten genes (*TUBB3*, *TRIM69*, *ZNFX1*, *VAV1*, *KCNN2*, *VGLL3*, *AK7*, *ARMC4*, *LRRC1*, and *CRIP1*). To understand the relationship between potential hub genes and survival status, the risk scores for these 21 genes were calculated (Fig. 5A), and from these scores we established a ten-gene risk model. The resulting heat map revealed that genes *ARMC4*, *LRRC1*, and *TUBB3* were overexpressed in the high-risk group, and that genes *AK7* and *CKNN2* were overexpressed in the low-risk group (Fig. 5B). Using the TCGA sarcoma dataset values, a survival status map was plotted to demonstrate the status for each sample (Fig. 5C). The risk scores, gene expression differences, and survival status for the ten hub genes, highlighted that AS events had the potential for prognosis predictions in sarcoma. We then constructed a multi-factor survival model and used it to analyze the variable-splicing events and expression-profile levels of these ten hub genes. The prognosis classifications are shown in Fig. 5D–G. This model demonstrated good prognostic classifications based on the RNA-seq and SpliceSeq data, and the AUC values were very high, suggesting that the ten genes of this model could be used as prognostic biomarkers for sarcoma.

### Hub gene expression and overall survival of patients with sarcoma

**Figure 6:**
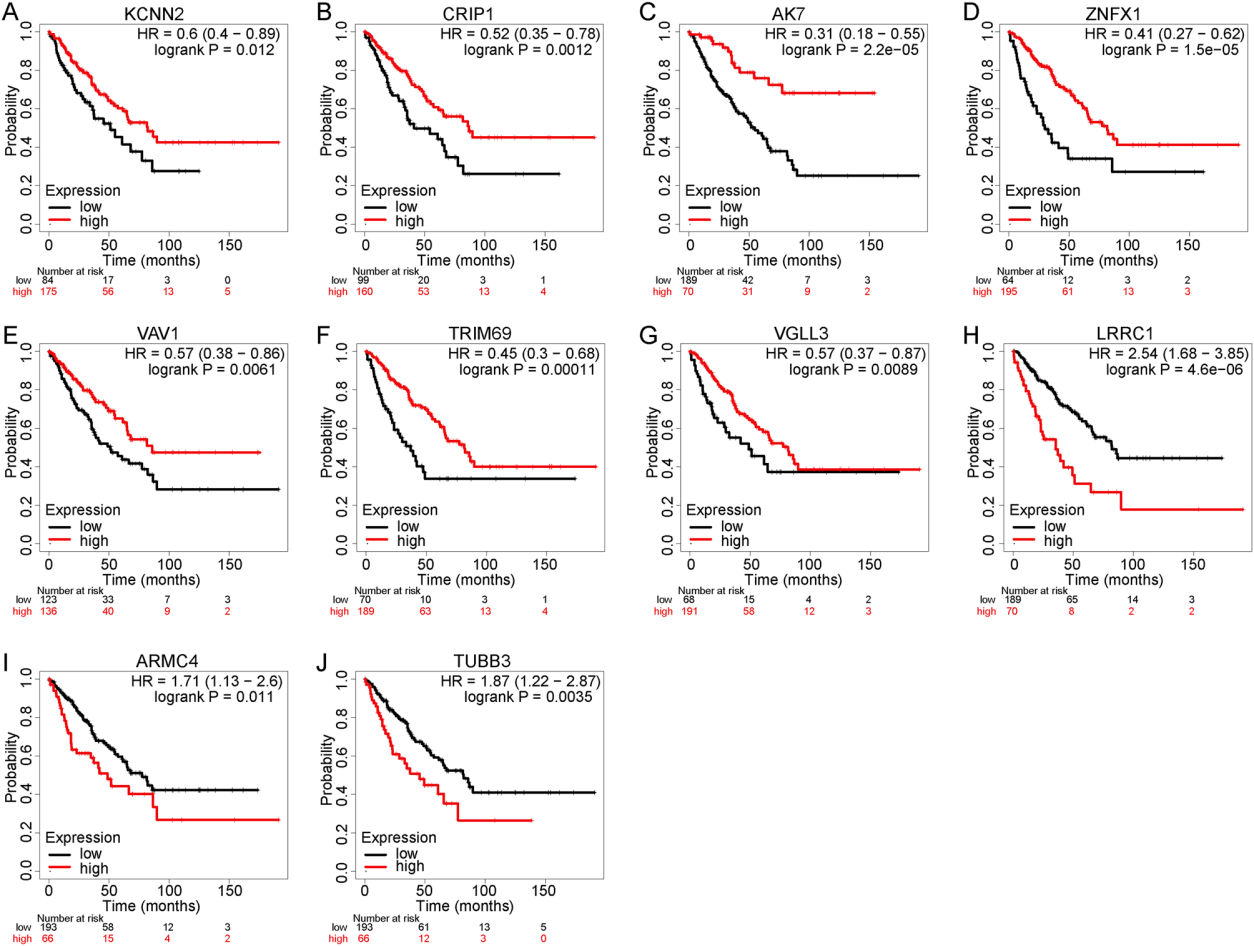
The association between the expression levels of the ten hub genes and overall survival of patients with sarcoma. (**A**–**G**) The upregulated expression levels of *KCNN2*, *CRIP1*, *AK7*, *ZNFX1*, *VAV1*, *TRIM69*, and *VGLL3* were significantly associated with a better overall patient survival for sarcoma. (**H**–**J**) The upregulated expressions of *LRRC1*, *ARMC4*, and *TUBB3* were significantly associated with poor prognoses in sarcoma patients.

These ten hub genes also had predictive value for patient survival. As shown in the Fig. 6A–G, the elevated expression levels of *KCNN2*, *CRIP1*, *AK7*, *ZNFX1*, *VAV1*, *TRIM69*, and *VGLL3* were significantly associated with a better overall patient survival. However, the Kaplan–Meier analyses showed that the increased expressions of *LRRC1*, *ARMC4*, and *TUBB3* were significantly associated with unfavorable patient prognoses (Fig. 6H–J). Overall, the findings demonstrate that these hub genes may serve as novel biomarkers for use in prognostic predictions.

## Discussion

AS events frequently affect RNA binding, targeting specific RNA sequences or motifs which play important roles in gene expression regulation, cellular growth, development, tissue homeostasis, and RNA-species diversity^8,14^. AS events can be detected at the transcript level, using microarrays and RNA-seq data, and accumulating evidence indicates that aberrant RNA splicing patterns are associated with both the growth and progression of tumors^23,24^. Survival-related splicing factors are also important for AS events, as events that are favorable to overall survival were reported to be negatively associated with splicing factors in soft tissue sarcoma^25^. In Ewing sarcomas, hnRNPM-dependent AS promoted drug resistance and drove resistance to the inhibition of the PI3K/AKT/mTOR pathway^26^. It was reported that the majority of survival-associated AS events were also poor prognostic markers for sarcoma^27^. EWS-FLI1 was reported to play a crucial role in the AS-regulation process of Ewing sarcoma^28^. Consistent with previous studies, our analysis of the relationship between AS events and sarcoma-patient prognoses revealed that AS events were involved in sarcoma progression and may be potential predictors for sarcoma prognoses.

In this study we also identified ten prognosis-related genes (*TUBB3*, *TRIM69*, *ZNFX1*, *VAV1*, *KCNN2*, *VGLL3*, *AK7*, *ARMC4*, *LRRC1*, and *CRIP1*) closely associated with the occurrence and development of sarcoma. *TUBB3*, which codes for a microtubule protein, was overexpressed and linked to poor prognoses in a variety of cancers^29,30^. The function of *TRIM69*, a member of the tripartite motif (TRIM) family, has been reported to inhibit virus replication through a transcription-inhibition mechanism that prevented the synthesis of viral messenger RNAs^31^. *ZNFX1*, a novel lncRNA that has been shown to regulate cell proliferation, the cell cycle, cell migration, and cell invasion, may be a promising biomarker to predict poor prognoses in many cancers^32–34^. The ten hub genes were also involved with cancer initiation and progression, correlating with progression^35^. *VAV1* has been reported to play a crucial role in the progression of human cancer, and *AK7* expression has been positively correlated with malignant-cell proliferation in both acute lymphoblastic leukemia and Burkitt's B cell lymphoma^36^. *ARMC4*, an axonemal protein necessary for proper targeting, has also been identified among the novel genes associated with tumorigenesis in colorectal cancer^37^. *LRRC1*,a putative cell-polarity regulator, was significantly upregulated and considered to be a potential oncogene in hepatocellular carcinoma^38^. *CRIP1* has been reported to be overexpressed in many cancer tissues and is considered to be an oncogene in tumor development and progression^39^.

The present ten gene-based model for prognostic predictions, focused on changes in AS events and changes in the expression levels of AS-related genes. The multivariate survival model assessed AS-related survival times for these ten hub genes, and successfully classified sarcoma prognoses. All of these hub genes are involved in a variety of oncogenesis and cancer progression functions, which indicates that this model may serve as a good predictor of survival prognoses for patients with sarcoma.

The present study verified that prognosis-associated AS events were ideal to construct a prognosis-prediction model. Using AS-related gene expression levels, we also identified the ten hub genes most relevant for this model. This novel model shows potential to contribute to both clinical and therapeutic approaches to better treat sarcoma.

## Materials and methods

### Data downloading and preprocessing

RNA-seq data from sarcoma AS events were downloaded from the SpliceSeq database (https://bioinformatics.mdanderson.org/TCGASpliceSeq/) compiled from TCGA. Data from a total of 261 samples were collected from the TCGA database (https://cancergenome.nih.gov/), including two normal tissue samples, and the RNA-seq gene expression profiles were from sarcomas and para-cancerous tissues. All represented patients had well-documented clinical information and follow-up data. The gene expression data in fragments per kilobase of exon model per million reads mapped (FPKM) were transformed into transcripts per million (TPM) values.

The corresponding gene profiles from the human genome, version GRCh38.p2 from GENECODE, were processed using gene ID conversion. In total, 236 protein-coding genes common to both the SpliceSeq and RNA-seq cohorts were identified. Lastly, we obtained the expression data for 19,754 genes for further analyses.

### Analyses of RNA-seq data and AS events

SpliceSeq is a java application for the visualization and quantitation of splice junctions and exon proportions included in TCGA data. We defined seven AS subtypes for sarcomas: exon skip (ES), mutually exclusive exons (ME), retained intron (RI), alternate promoter (AP), alternate terminator (AT), alternate donor site (AD), and alternate acceptor site (AA).

### Selection of survival-related AS events

Analyses of patient survival data were made using the survival package in R software (http://cran.r-project.org/package=survival), and the level of significance was set at *P* < 0.05.

### Network analysis for AS-related genes

For the analysis of gene-network interactions, we used the STRING database (http://string-db.org/), and applied a threshold score > 0.4. Data visualization was performed using Cytoscape (https://cytoscape.org/).

### A prognosis model based on ten hub genes

We performed a multivariate analysis of the gene expression data using the Cox proportional hazard-regression model. We identified the ten AS-related genes that demonstrated the most significant expression-profile changes, and then used these hub genes to create a prognostic model and for survival analyses. In addition, we determined the correlations between the expression profiles of these ten hub genes and overall patient survival based on Kaplan–Meier plots (https://kmplot.com/).

### Correlations between AS subtypes and prognoses

We systematically explored patient prognoses associated with AS-subtype events and calculated AS-event distributions. We then determined the correlations between prognosis-related genes and these AS-event distributions.

### Biological functions analysis

To determine the functions of the prognostic genes that were significantly related to AS events, we used KOBAS 3.0 (http://kobas.cbi.pku.edu.cn/), a web-based tool for gene/protein functional annotation and pathway-enrichment analysis.

### Prognostic model construction

Characteristic genes were selected as being significantly prognostic with Pearson correlation coefficients of more than 0.5 or less than − 0.5. To construct our prognostic model, we used these prognostic genes and the frequencies of AS events, to develop a sarcoma prognostic index.

### An analysis of prognosis-related genes

To determine correlations between levels of gene transcription and variable-splicing prognoses, we used a univariate analysis of AS-sequence profiles using TCGA RNA-seq data for associated genes to determine the impact of AS events on sarcoma prognosis.

## Data Availability

All of the data involved in this study are available in the public databases which are listed in the “Materials and methods” section.
